# Duodenal Ferroportin Is Up-Regulated in Patients with Chronic Hepatitis C

**DOI:** 10.1371/journal.pone.0110658

**Published:** 2014-10-20

**Authors:** Lanqing Ma, Tong Zou, Yuping Yuan, Jiajun Lv, Xiangqian Dong, Gang Yang, Yunzhen Zhu, Juan Luo, Zhigang Zhang, Jiefu Yang

**Affiliations:** 1 Department of Digestive Diseases, The First Affiliated Hospital, Kunming Medical University, Kunming, Yunnan, China; 2 Department of Cardiology, Beijing Hospital, Ministry of Health, Beijing, China; 3 State Key Laboratory of Genetic Resources and Evolution, Kunming Institute of Zoology, CAS, Kunming, Yunnan, China; University of Malaya, Malaysia

## Abstract

Hepatitis C virus (HCV) infection is a leading cause of liver-related mortality. Chronic hepatitis C (CHC) is frequently associated with disturbances in iron homeostasis, with serum iron and hepatic iron stores being elevated. Accumulating evidence indicates that chronic HCV infection suppresses expression of hepatic hepcidin, a key mediator of iron homeostasis, leading to iron overload conditions. Since hepcidin mediates degradation of ferroportin, a basolateral transporter involved in the release of iron from cells, diminished hepcidin expression probably leads to up-regulation of ferroportin-1 (Fpn1) in patients with CHC. In this study, we determined the protein levels of duodenal Fpn1, and found that its expression was significantly up-regulated in patients with CHC. The expression of duodenal Fpn1 is negatively correlated with mRNA levels of hepcidin, and positively correlated with serum iron parameters. Although iron is a critical factor for growth of a variety of pathogenic bacteria, our results suggest that iron overload in blood does not increase the infection rate of bacteria in patients with CHC.

## Introduction

As a co-factor for heme and non-heme iron proteins, iron is an important element for all living organisms; however, excess iron can be toxic for the organism [1]. Therefore, iron metabolism must be tightly regulated. Absorption of dietary iron begins with its transport through the plasma membrane by the divalent metal transporter 1 (DMT1), the major iron transporter in the duodenum [2], [3]. After entering the cytoplasm of enterocytes, iron is stored in ferritin or transferred to the portal blood by the basolateral exporter ferroportin-1 (Fpn1). Fpn-1, a principal iron exporter, plays in transporting iron from the duodenum to the serum, and in iron recycling from reticuloendothelial macrophages into the circulation, respectively [4]. Hepcidin, the key mediator of iron homeostasis produced by liver, negatively controls Fpn1 expression levels by a posttranslational mechanism [5]. Hepcidin mediates internalization and induces degradation of Fpn1 in enterocytes and reticuloendothelial macrophages, thus suppressing duodenal iron absorption and macrophage iron release. The expression of hepcidin is significantly up-regulated by iron overload and inflammation [6], [7], and down-regulated in response to erythropoiesis, iron deficiency, and hypoxia [8].

Iron deposition is a pathophysiologic feature during chronic hepatitis C virus (HCV) infection [9]. Excess iron accumulation can be highly toxic, and contributes to liver injury by inducing oxidative stress [10]. Accumulating evidence indicates that both expression of hepcidin mRNA in liver and serum levels of hepcidin are decreased in patients with chronic hepatitis C (CHC) [11]–[13]. Although the precise mechanism underlying inhibition of hepcidin expression by HCV is not fully understood, production of reactive oxygen species (ROS) is believed to be involved in the process [14], [15]. In vitro experiments demonstrate that ROS induced by HCV proteins suppress the expression of hepcidin by inhibiting binding activity of two positive regulators, C/EBPβ and STAT3, and stabilizing the expression of two negative hepcidin regulators, HIF1α and HIF2α in human hepatoma cell lines [14]. In HCV transgenic mice, ROS induced by HCV increase hepatic expression of CHOP and subsequently reduce DNA binding activity of C/EBPα, which leads to down-regulation of hepcidin mRNA levels. Decreased hepcidin expression enhances ferroportin expression in the duodenum and macrophages, resulting in an increase in duodenal iron transport and a decrease in macrophage iron release [15].

While HCV transgenic mice exhibit high levels of duodenal ferroportin [15], not much is known concerning the expression of ferroportin in patients with CHC. In this report, we evaluated the protein expression of Fpn-1 in duodenum biopsy samples from patients with CHC. Since bacterial pathogens need iron to replicate and survive [16]–[18], we also investigated whether increased iron levels in blood could influence bacterial communities in patients with CHC.

## Materials and Methods

### Liver and duodenum samples

We recruited 131 patients with CHC and 47 patients with nonalcoholic fatty liver disease (NAFLD) (Control group), who were hospitalized between May 2011 and May 2013 at The First Affiliated Hospital, Kunming Medical University, China. Patients with CHC were eligible for inclusion in the study if they were HCV seropositive, had detectable HCV RNA that liver biopsy was performed. Exclusion criteria were viral hepatitis other than hepatitis C, hepatic cirrhosis, alcoholic or drug-induced liver injury, hepatocellular carcinoma, serious cardiopulmonary and renal disease, acute inflammatory disorders, and upper gastrointestinal bleeding. The diagnosis of chronic HCV infection was based on the measurement of serum anti-HCV antibody (Auto delfia1235, PerkinElmer, Inc, Waltham, MA, USA) and HCV RNA (Freedom Evolyzer-z, Tecan Group Ltd., Männedorf, Switzerland). NAFLD is diagnosed according to the following three criteria: non-alcoholic, detection of steatosis ultrasonography and liver biopsy, and appropriate exclusion of other liver diseases [19]. Of these patients, 43 patients with HCV and 22 with NAFLD suffered from persistent or recurrent pain or discomfort centred in the upper abdomen. After clinical examination and routine biochemistry, these patients underwent upper gastrointestinal endoscopy. Exclusion criteria were the presence of duodenal duodenitis and gastroduodenal ulcer on endoscopy. During the upper gastrointestinal endoscopy, duodenal biopsies were taken from 15 patients with HVC and 12 patients with NAFLD, respectively. Furthermore, liver samples were obtained from these 27 patients by needle biopsy for diagnosis of chronic liver diseases. Thus, 27 liver and duodenum samples were included in this study. Written informed consent was obtained from each patient included in the study. The study was approved by the Kunming Medical University Ethics Committee and carried out according to the guidelines.

### Assays for inflammatory factors, transaminases and iron parameters

Blood samples of all these subjects were collected in the morning under fasting conditions, and sera were separated immediately after blood collection and stored at −80°C until use. Serum levels of tumor necrosis factor (TNF-α) and interleukin-6 (IL-6) were determined using the commercially available ELISA kits (R&D Systems, Minneapolis, MN). The assays were performed according to the manufacturer’s protocols. Serum iron status, alanine aminotransferase (ALT), aspartate aminotransferase (AST), and bilirubin were determined using an automated Olympus AU5400 biochemistry analyzer (Olympus Corp., Tokyo, Japan). Hemoglobin levels and red blood cell count in blood were determined by ADVIA 120 hematology analyzer (Siemens, Healthcare Diagnostics Inc., Deerfield, IL, USA).

### Immunohistochemistry and prussian blue staining

After liver and duodenal biopsies were fixed in 10% buffered formalin, these tissues were embedded in paraffin, and sectioned at 5 µm in thickness. For immunohistochemical analysis, the sections were blocked by 3% BSA-PBS solution at room temperature for 1 h. The sections were then stained with monoclonal anti-prohepcidin (Santa Cruz Biotechnology, Santa Cruz, CA; 1∶250 dilution) or goat anti-Fpn1 antibodies (Santa Cruz Biotechnology; 1∶250 dilution) at 4°C overnight, respectively. After washed with PBS three times for 10 min at room temperature, the sections were incubated with HRP-conjugated anti-mouse or goat IgG (Boster Biol Tech, Wuhan, China) for 60 min at room temperature. The substrate solution diaminobenzidine (Tiangen, Beijing, China) was added until the desired intensity of color was developed. Prussian blue staining for iron was performed using a Prussian blue staining kit (Polysciences, Inc. Warrington PA, USA) according to the manufacturer’s protocol.

### Real-time PCR

Total RNA was extracted from liver tissue using Trizol reagent (Invitrogen, Carlsbad, USA). cDNAs were generated by reverse transcription with SuperScript II (Invitrogen). Real-time PCR was performed with the ABI Prism 7000 Sequence Detection System (Applied Biosystems, Foster City, USA) using SYBR Premix-Ex TagTM (TaKaRa, Dalian, China). The primers used were: hepcidin, 5′- CTG CAA CCC CAG GAC AGA G -3′ (forward), 5′- GGA ATA AAT AAG GAA GGG AGG GG -3′ (reverse) [20]; Fpn1, 5′- CTA CTT GGG GAG ATC GGA TGT -3′ (forward), 5′- CTG GGC CAC TTT AAG TCT AGC -3′ (reverse) [21]; and GAPDH, 5′- ACT TTG GTA TCG TGG AAG GAC TCA -3′ (forward); 5′- GTA GAG GCA GGG ATG ATG TTC TG -3′ (reverse).

### Western blotting

After duodenal samples were homogenized in liquid nitrogen, the homogenate was lysed on ice for 30 minutes in lysis buffer (BioTeKe, Beijing, China). The lysates (25 µg of total protein) were loaded per well and separated on a 10% SDS-polyacrylamide gel. Proteins were then transferred onto a polyvinylidene difluoride membrane. Primary antibodies were goat anti-Fpn1 antibodies (Santa Cruz Biotechnology, 1∶2500 dilution). The secondary antibody was a peroxidase-coupled anti-goat IgG (Boster Biol Tech). The membrane was exposed to Pierce® ECL Western Blotting Substrate (Thermo Scientific, Rockford, lL), and the film was developed.

### Sample preparation, pyrosequencing and bioinformatics

Genomic DNAs of bacteria in serum were extracted, the 16S ribosomal RNA (rRNA) gene V1–V2 region was amplified by PCR, and the amplicons was sequenced on a 454 Genome Sequencer FLX Titanium platform, as described in our recent studies [22], [23]. To eliminate artificial contaminations, potential presence of bacteria in all reagents for DNA extract and PCR were detected by amplifying bacterial 16S rRNA using PCR methods as described above. Sequencing reads were quality filtered, OTU clustered, ChimeraSlayer filtered and further analyzed using the QIIME pipeline and RDP-classifier. All OTUs found in 50% of blood samples were retained for the following further analyses. PLS-DA plotting analysis of samples based on microbiota analysis was performed using METAGENassist, a comprehensive web server for comparative metagenomics.

### Statistical analysis

Statistical analysis was performed using SigmaPlot 12.0 (Systat Software Inc., London, UK). General characteristics were expressed as median and mean or percentages. Two samples comparisons were performed using t-test (parametric) or Mann-Whitney rank sum test (non-parametric). Correlation analyses between two samples were performed using Spearman’s rank correlation. Statistical significance was set at *P*<0.05.

## Results

### Clinical characteristics of the patients

The characteristics of patients with CHC and without HCV infection (the control group) are shown in Table 1. There was no statistical difference in age and sex distribution in the two groups. In patients with CHC, the mean values of HCV RNA was 990±205 kIU/ml. Serum levels of ALT, AST, IL-6, and TNFα in patients with CHC were significantly higher than those in the control group. In contrast, patients with CHC had similar red blood cell counts, hemoglobin, and hematocrit levels, compared to the control group.

**Table 1:** Clinical characteristics of patients in this study.

	HCV(+) patients	HCV(−) patients	P value
Age, y	50.6±14.2 (31∼67)	56.7±13.1 (42∼71)	>0.05
Gender (M/F)	9/6	8/4	>0.05
ALT (IU/L)	66.5±25.6	42.2±17.3	<0.05
AST (IU/L)	60.6±31.4	36.7±15.1	<0.05
Hematocrit (%)	40.2±4.8	42.1±5.3	>0.05
RBC (10^4^/mm^3^)	427±91	465±88	>0.05
Hemoglobin (g/L)	12.8±1.7	13.5±1.8	>0.05
Serum ferritin (ng/ml)	208±144	106±74	<0.01
Transferrin saturation (%)	56±22.4	24.5±18.5	<0.001
IL-6 (pg/ml)	5.4±4.1	2.5±1.4	<0.01
TNFα (pg/ml)	11.4±6.1	6.8±4.2	<0.05

**Figure 1:**
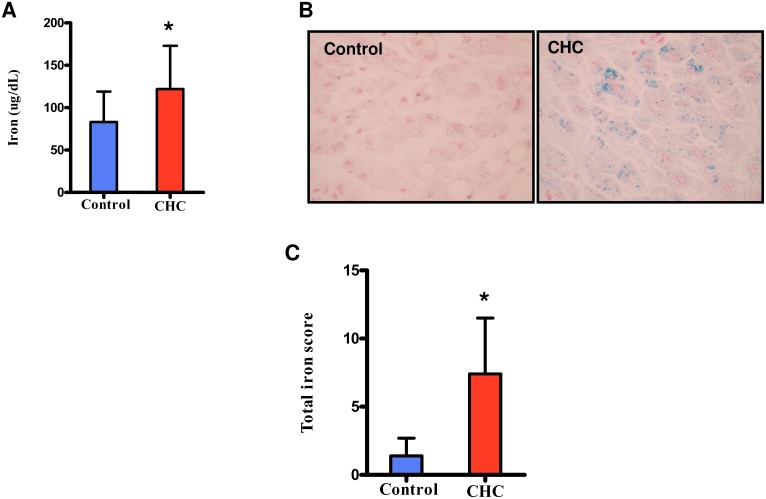
Iron concentrations in serum, and iron localization in the liver. CHC (*N* = 15) and Control (*N* = 12). (A) Serum levels of iron. (B) Iron in liver sections with Prussian blue staining. (C) Total iron score. *P<0.05 relative to control group.

It has been shown that patients with CHC have higher levels of serum iron [9], [11]. Consistent with these observations, we found that serum levels of iron in patients with CHC in this study were markedly higher than those in the control group (Fig. 1A). Meanwhile, serum levels of transferrin saturation, and ferritin levels in patients with CHC were significantly higher than those in the control group (Table 1). In addition, moderate iron deposition in the liver was observed in patients with CHC (Fig. 1B), and TIS scores were much higher in patients with CHC than in the control group (Fig. 1C).

### The expression of hepcidin in liver is reduced in patients with CHC

**Figure 2:**
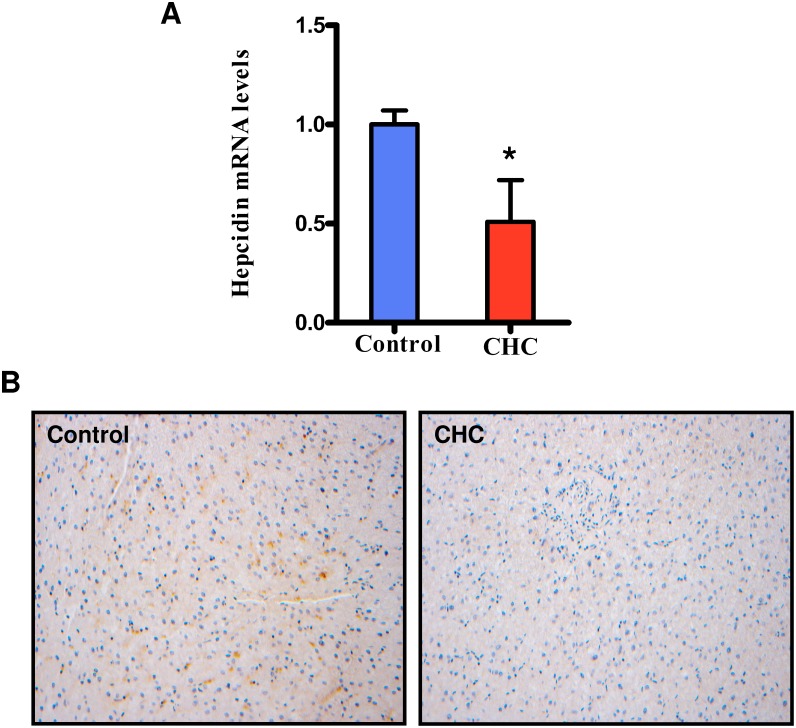
The expression of hepcidin in the liver. CHC (*N* = 15) and Control (*N* = 12). (A) mRNA levels of hepcidin determined by qPCR. (B) The protein expression of prohepcidin determined by immunohistochemistry. *P<0.05 relative to control group.

Next, we determined the mRNA levels of hepcidin by qPCR in all subjects. As shown in Fig. 2A, the mRNA levels of hepcidin in the patients with CHC were significantly lower than those in the control group. Meanwhile, we measured the protein levels of prohepcidin in the liver by immunohistochemical staining. Patients with CHC exhibited a decrease in the expression of hepatic prohepicidin, compared with the control group.

### Expression of duodenal Fpn1 is up-regulation in patients with CHC

**Figure 3:**
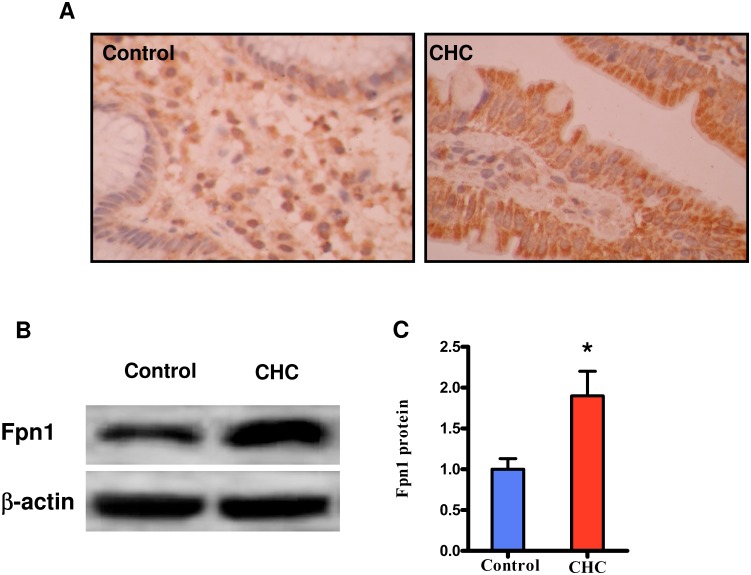
The protein expression of duodenal Fpn1. CHC (*N* = 15) and Control (*N* = 12). The protein expression of duodenal Fpn1 determined by immunohistochemistry (A), and Western blot (B), respectively. (C) Quantification of protein levels by Western blot in the two groups. *P<0.05 relative to control group.

Hepcidin binds to the iron exporter ferroportin, leading to its internalization and degradation [5]. Thus, reduced hepcidin levels probably results in an increase in the levels of Fpn1 in the duodenum. To test this hypothesis, we measured the expression of Fpn1 in the duodenum by immunohistochemical analysis. We found that the protein expression of Fpn1 in the duodenum was significantly higher in patients with CHC than in the control group (Fig. 3A). To further confirm these results, the protein expression of Fpn1 in the duodenum was detected by western blotting. As shown in Fig. 3B and C, the protein levels of duodenal Fpn1 were markedly up-regulated in patients with CHC, compared with those in the control group. In contrast, the mRNA levels of Fpn1 the patients with CHC were comparable to those in the control group (data not shown).

### Correlation between duodenal Fpn1 levels and clinical parameters

We next examined correlation of duodenal Fpn1 protein with inflammatory factors and iron parameters (Table 2). We found that hemoglobin concentrations, red blood cell counts, hematocrit, and the serum levels of ALT and AST, were not correlated with duodenal Fpn1 protein levels. As a potent stimulator of prohepcidin expression in liver [7], [24], IL-6 in serum was significantly increased in patients with CHC (Table 1). However, a decrease in the expression of hepatic prohepicidin was observed in patients with CHC. Thus, it is unlikely that the expression of prohepcidin is regulated by inflammation in CHC. Furthermore, we found that there were no significant correlations between duodenal Fpn1 protein and inflammation factors (IL-6, and TNFα) in patients with CHC. In contrast, Fpn1 protein levels were negatively correlated with hepcidin mRNA levels. Finally, we found that there were significant correlations between Fpn1 protein levels and serum iron levels. Taken together, these results suggest that elevated duodenal Fpn1 protein levels are probably due to reduced hepcidin expression, leading to an increase in serum levels of iron.

**Table 2:** Correlations between clinical findings and Fpn1 protein levels in patients with CHC.

	r	P value
ALT (IU/L)	0.153	0.0912
AST (IU/L)	0.168	0.1340
Hematocrit (%)	0.112	0.1781
RBC (10^4^/mm^3^)	0.154	0.0898
Hemoglobin (g/L)	0.177	0.0612
Serum ferritin (ng/ml)	0.528	<0.0001
Transferrin saturation (%)	0.368	0.0015
Serum iron (µg/dL)	0.288	0.0236
TIS	0.465	<0.0001
Hepcidin RNA levels	−0.381	0.0011
IL-6 (pg/ml)	0.179	0.0781
TNFα (pg/ml)	0.118	0.1390

### Iron overload in blood does not increase the risk of bacterial infection in patients with CHC

Many species of pathogenic bacteria require iron for growth as iron acquisition proteins are virulence factors for these pathogens [16]–[18]. As iron overload in blood occurs in patients with CHC, we hypothesized that increased iron levels in blood could increase the infection rate of pathogenic bacteria in patients with CHC. To test this idea, we determined the composition of bacterial communities in blood using high-throughput 454 pyrosequencing. In total, we collected blood samples from 12 control patients and 15 patients with CHC, for analysis of the bacterial 16S ribosomal RNA (rRNA) genes. The variable regions (V1–V2) of the bacterial rRNA 16S genes were amplified by PCR using a primer set with a unique 10-nt barcode. Three blood samples from the CHC group and three blood samples from the control group were excluded from the following analysis due to sequences less than 500, which possibly resulted from sequencing bias. For the remaining 21 samples, we obtained a dataset consisting of 31, 205 high-quality 16S rRNA gene sequences with an average of 1486±188 (S.E.) sequences per sample (Table 3). In this study, the most notable contamination was derived from the reagents for DNA extract and PCR. But our PCR-based bacteria detections for all these reagents were negative.

**Table 3:** 454 data summary.

	CHC Group (n = 12)	Control Group (n = 9)	Total (n = 21)
**Sequence number**	1960±248	854±79	1486±188
**OTUs** a	73±7	64±6	69±5
**Shannon** b	2.21±0.13	2.57±0.14	2.37±0.10
**Simpson** b	0.53±0.03	0.60±0.02	0.56±0.02

a OTU was equal to bacterial species level according to 97% sequence identity.

b Bacterial diversity Index.

From the dataset, we identified a total of 265 operational taxonomic units (OTUs) (detected from at least two blood samples) based on the conventional criterion of 97% sequence similarity (equal to species level), with 64±6 (n = 9) OTUs per sample in blood from the control group, 73±7 (n = 12) OTUs per sample in blood from the CHC group (Table 3). Furthermore, we found 14 of 16 dominant OTUs can be matched to known bacterial species according to 97% sequence similarity, which accounted to about 90% of all bacterial 16S rDNA sequences (Table 4).

**Table 4:** Identification of 14 dominant bacterial species and their abundance distribution in CHC and control groups.

OTUs ID	CHC Group#(n = 12)	Control Group#(n = 9)	Similar Species	Genbank Accession	Identity
**OTU01**	66.10±2.97%	60.70±2.16%	*Pseudomonas aeruginosa* DK2	NC_018080	99%
**OTU02** ***	7.13±1.15%	12.80±0.65%	*Delftia acidovorans* SPH-1	NC_010002	99%
**OTU03**	5.52±2.29%	2.15±1.75%	*Pseudomonas fluorescens* A506	NC_017911	99%
**OTU04**	3.48±0.22%	3.80±0.33%	*Pseudomonas aeruginosa* DK2	NC_018080	99%
**OTU05**	2.49±0.21%	2.89±0.13%	*Pseudomonas aeruginosa* DK2	NC_018080	98%
**OTU06**	1.75±0.52%	0.81±0.10%	*Escherichia coli* str. K-12 substr. W3110	NC_007779	99%
**OTU07**	1.11±0.10%	1.16±0.09%	*Pseudomonas aeruginosa* DK2	NC_018080	99%
**OTU08**	1.36±0.60%	0.42±0.42%	*Rhodococcus qingshengii* strain djl-6	NR_043535	99%
**OTU09**	0.51±0.06%	0.68±0.10%	*Pseudomonas aeruginosa* DK2	NC_018080	99%
**OTU10**	0.50±0.06%	0.68±0.07%	*Pseudomonas aeruginosa* DK2	NC_018080	99%
**OTU11**	0.60±0.19%	0.26±0.06%	*Escherichia coli* O26:H11 str. 11368	NC_013361	99%
**OTU12**	0.46±0.20%	0.25±0.21%	*Phyllobacterium myrsinacearum*	AB681132	99%
**OTU13**	0.33±0.11%	0.41±0.13%	*Propionibacterium acnes* 6609	NC_017535	99%
**OTU14**	0.36±0.07%	0.36±0.07%	*Pseudomonas aeruginosa* DK2	NC_018080	99%

# Relative abundance given by mean ± SE.

***P = <0.001 (t-test).

**Figure 4:**
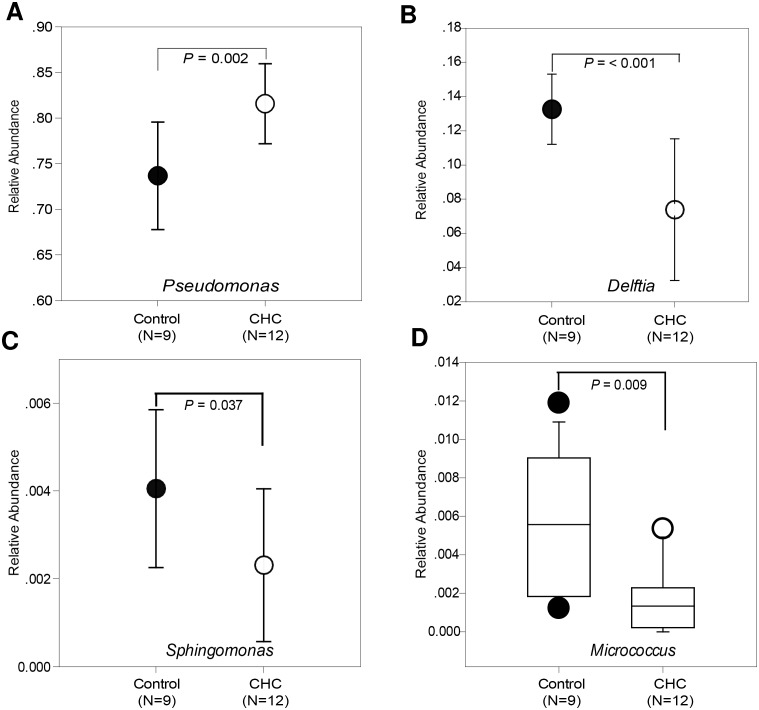
Bacterial genera comparison of blood samples between the CHC (*N* = 12) and control (*N* = 9) groups. (A) *Pseudomonas*; (B) *Delftia*; (C) *Sphingomonas*; (D) *Micrococcus*.

We observed no difference in bacterial community diversities between the control and CHC groups (evenness by Shannon and Simpson indexes in Table 3). However, we found that the bacteria genus *Pseudomonas* in the CHC group was significantly higher than that in control group (81.60±4.38% versus 73.70±5.90%) (Fig. 4A). We also discovered a significant decrease in the abundance of three rare bacterial genera *Delftia*, *Sphingomonas,* and *Micrococcus* in the CHC group, compared with the control group (Fig. 4C–D).

## Discussion

It has been reported that the serum levels of hepcidin and mRNA levels of hepcidin in the liver in patients with CHC are significantly lower than normal subjects or patients without HCV infection [11]–[13]. Consistent with these observations, our results confirm that patients with CHC have low levels of hepcidin in serum and prohepicidin in the liver. The expression of hepcidin is significantly induced by iron overload and inflammation [6], [7] and downregulated in response to low iron levels, erythropoiesis, and hypoxia [8]. However, high serum levels of iron and IL-6, and iron deposit in liver does not result in high levels of hepcidin in patients with CHC. The fact that ROS induced by HCV proteins inhibits the expression of hepcidin [14], [15] strongly suggests that the ROS-mediated signaling overcomes inflammatory and iron-sensing pathways in hepcidin regulation in patients with CHC. Thus, elevated iron levels in serum are largely attributable to reduced hepcidin expression in liver.

As a peptide hormone, hepcidin function as a regulator of iron homeostasis by inhibiting the iron transporter ferroportin [5]. Nishina et al has previously reported that ferroportin expression in the duodenum and macrophages is significantly up-regulated in HCV transgenic mice [15]. However, the HCV transgenic mouse model used by Nishina et al is characterized by a lack of hepatic inflammation. Therefore, the animal model is different from patients with CHC whose serum levels of IL-6 are elevated [25]. In an attempt to shed light on the pathogenesis of iron overload in CHC, we determined the expression of duodenal ferroportin. Our results have demonstrated that the protein levels of Fpn1 in the duodenum increase in patients with CHC. This observation is consistent with presently elucidated hepcidin functions. When serum levels of hepcidin are reduced, Fpn1 in the duodenum is elevated. Increased intestinal iron absorption, at least in part, account for high levels of serum iron in patients with CHC. However, we do not exclude the possibility that high levels of serum iron are due to an increase in the protein expression of Fpn1 in reticuloendothelial macrophages in patients with CHC.

It has been well-established that CHC is associated with elevated serum iron indices and hepatic iron stores [9], [11]. Interestingly, in vitro experiments demonstrate that iron blocks HCV replication in hepatoma cell lines [26], [27]. These findings suggest that iron can exert antiviral effects. On the other hand, pathogenic bacteria require iron for survival [17], [18]. Thus, inflammatory hypoferremia has been regarded as an antibacterial defense mechanism to limit bacterial iron availability [16]–[18]. Currently, the origin of blood-associated bacteria remains unclear. Using multiple methods, such as dark-field microscopy, fluorescent *in* *situ* hybridization, and PCR-based analysis, several studies have demonstrated that a variety of bacteria exist in the normal human blood [28]–[30]. In this study, our results demonstrate that bacterial genera from control and CHC groups exhibit a similar pattern, suggesting that an overabundance of iron does not alter the bacterial communities in patients with CHC. It has been reported that *Helicobacter* 16S rDNA is detected in more than 60.0% of liver samples from patients with HCV using PCR methods [31]. However, we failed to detect the presence of *Helicobacter* species in the blood samples from patients with CHC. Thus, coinfection with *Helicobacter* species in patients with CHC does not seem to be common. Consistent with previous observations [28]–[30], our study indicate that *Pseudomonas* is the most abundant bacterial genus in blood. Although *Pseudomonas* genus in the CHC group are significantly higher than those in control group (Figure 4), all dominant species within this genus have no significant difference between the CHC and control groups (Table 4). These results indicate that the member of *Pseudomonas* genus may not have any clinical significance related to HCV infection. Consistent with significant decrease of *Delftia* genus in the CHC group, relative abundance of its representative species (*Delftia acidovorans* SPH-1) in the CHC group is significantly lower than that in the control group (Table 4). However, whether this bacterial species has the clinical significance related to HCV infection remains unclear. Taken together, this study suggests that iron overload in blood does not increase potential risk of bacterial infection in patients with CHC.
